# Identifying miR-431-5p as a promising candidate biomarker for early-onset severe preeclampsia through integrated bioinformatic and clinical analyses: a retrospective study

**DOI:** 10.7717/peerj.21591

**Published:** 2026-08-11

**Authors:** Jianxin Zhang, Hongwei Li, Yu Yan, Xiaoxiao Li, Linlin Zhang, Yangnan Ding, Jun Ma, Enwu Yuan, Xin Zhao

**Affiliations:** 1 Zhengzhou Key Laboratory for In Vitro Diagnosis of Hypertensive Disorders of Pregnancy, Zhengzhou, Henan, China; 2 The Third Affiliated Hospital of Zhengzhou University, Zhengzhou, Henan, China; 3 Department of Laboratory Medicine, The Third Affiliated Hospital of Zhengzhou University, Zhengzhou, Henan, China; 4 Tianjian Laboratory of Advanced Biomedical Sciences, Institute of Advanced Biomedical Sciences, Zhengzhou University, Zhengzhou, Henan, China

**Keywords:** Early-onset severe preeclampsia, miR-431-5p, Biomarker, Bioinformatics

## Abstract

**Background:**

Early-onset severe preeclampsia (EOSPE) is a serious pregnancy complication associated with adverse maternal and neonatal outcomes. This study aimed to identify potential diagnostic biomarkers for EOSPE and to investigate their clinical significance.

**Methods:**

MicroRNAs (miRNAs) expression datasets from the Gene Expression Omnibus (GEO) database, specifically from placental tissue (GSE103542) and plasma (GSE234611), were screened to identify miRNAs consistently dysregulated in early-onset preeclampsia (EOPE) and to discover robust biomarker candidates. Weighted gene co-expression network analysis (WGCNA) was employed to identify key modules associated with preeclampsia (PE). The differentially expressed miRNAs (DEmiRNAs) were intersected with miRNAs from WGCNA-identified modules to prioritize candidates. Functional enrichment analysis predicted target genes and involved pathways. Clinical samples (serum and placental tissues from EOSPE patients and normal pregnant women) and a lipopolysaccharide (LPS)-induced preeclampsia rat model were utilized to validate the expression of DEmiRNAs *via* reverse transcription quantitative polymerase chain reaction (RT-qPCR). The diagnostic value of these miRNAs was evaluated using receiver operating characteristic (ROC) curve analysis, and their correlations with clinical parameters were assessed.

**Results:**

Bioinformatics analysis identified miR-431-5p as a key molecule, with its expression was significantly upregulated in both serum and placental tissues of patients with EOSPE. Circulating miR-431-5p demonstrated promising diagnostic potential for EOSPE (AUC = 0.803). Its expression level positively correlated with systolic blood pressure (r = 0.395, *P* = 0.017) and diastolic blood pressure (r = 0.41, *P* = 0.013), uric acid (r = 0.389, *P* = 0.02), lactate dehydrogenase (r = 0.399, *P* = 0.018), and the umbilical artery S/D ratio (r = 0.457, *P* = 0.01). Conversely, it negatively correlated with gestational age at delivery (r = −0.562, *P* < 0.001) and neonatal birth weight (r = −0.503, *P* = 0.02). Multivariable regression analysis showed that after adjusting for confounding factors, miR-431-5p remained significantly positively associated with the occurrence of EOSPE. Animal experiments confirmed similar upregulation of miR-431-5p in the LPS-induced preeclampsia rat model.

**Conclusions:**

In this study, miR-431-5p was significantly upregulated in EOSPE and demonstrated preliminary diagnostic potential. Its expression level was potentially associated with disease severity and adverse perinatal outcomes. These findings suggest that miR-431-5p may serve as a promising candidate biomarker for EOSPE.

## Introduction

Preeclampsia (PE) is a serious pregnancy complication of placental origin, with an incidence ranging from 5.6% to 9.4% (Liu et al., 2023). It is not only the second leading cause of maternal mortality but also a major contributor to perinatal mortality and birth defects, thereby posing a significant obstacle to achieving national targets for reducing maternal and infant mortality (Langston-Cox et al., 2021). PE typically manifests after 20 weeks of gestation, with clinical features including hypertension, proteinuria, and multi-organ dysfunction (Psilopatis et al., 2023). This complication directly accounts for approximately 16% of stillbirths and perinatal deaths and can lead to various short-term and long-term complications (Stone et al., 2021). Furthermore, PE significantly increases the long-term risk of future cardiovascular and metabolic diseases in both mothers and their offspring (Shen et al., 2025).

As an important subtype of hypertensive disorders of pregnancy, early-onset preeclampsia (EOPE) is defined as preeclampsia manifesting before 34 weeks of gestation. It is characterized by rapid progression, a high risk of organ involvement, and a propensity to progress to a more severe stage, namely early-onset severe preeclampsia (EOSPE). In addition to the basic features of early-onset disease, EOSPE presents with persistent severe hypertension, multi-system organ dysfunction, and related complications, significantly impacting maternal and fetal outcomes. Current research indicates that EOSPE is closely associated with placenta-originated disorders and can lead to a series of adverse outcomes, including fetal growth restriction, placental abruption, iatrogenic preterm birth, and perinatal mortality. It remains one of the most challenging conditions in obstetric clinical management (Gangadhar et al., 2023; Huang et al., 2022; Tomimatsu et al., 2019). However, the specific pathophysiological processes underlying EOSPE remain incompletely understood. Consequently, critical challenges persist in its early identification, risk assessment, and management, underscoring the urgent need for more effective strategies.

A simple, reliable, and effective biomarker for clinical use remains unavailable. Although the sFlt-1/PlGF ratio is widely used for preeclampsia prediction, its specificity for EOSPE may be limited, and it does not reliably distinguish between different PE subtypes. Clinical management remains largely passive; aside from symptomatically alleviating maternal conditions and promoting fetal development, timely pregnancy termination and fetal delivery constitute the only effective treatment strategy. Nevertheless, maternal and neonatal outcomes continue to be suboptimal (Huhn et al., 2020; Mészáros et al., 2024; Shi et al., 2022). Therefore, there is an urgent need to identify specific biomarkers for EOSPE to enable early diagnosis, intervention, and treatment, thereby reducing the incidence of severe complications and improving maternal and neonatal outcomes.

MicroRNAs (miRNAs) are single-stranded non-coding RNA molecules approximately 22 nucleotides in length (Feng et al., 2016). Although miRNAs do not encode proteins, they participate in the regulation of gene expression. miRNA regulatory networks play important roles in many physiological and pathological processes, including cell proliferation and adhesion, angiogenesis, and immune cell development (Pankiewicz et al., 2021). Previous research has demonstrated that miR-431-5p expression is elevated in placentas from patients with PE compared with normal controls (Yang & Meng, 2019). Functionally, overexpression of miR-431-5p was shown to inhibit ZEB1, consequently reducing the migration and invasion capabilities of trophoblast cells. Research has found that serum levels of miR-431-5p are higher in PE patients than in normal controls, and its expression is even greater in patients with severe preeclampsia (sPE) than in those with non-severe preeclampsia (no-sPE). However, for the specific high-risk subtype of EOSPE, existing domestic and international studies have not yet systematically reported the relevant role of miR-431-5p. In particular, there is a lack of comparative analysis of its expression profiles in two key sample types—serum and placental tissues—from EOSPE patients. Moreover, its association with EOSPE disease progression and its clinical diagnostic potential remain to be clearly elucidated. Consequently, the role of miR-431-5p in EOSPE pathogenesis and its potential as a diagnostic biomarker are still unclear, highlighting the need for research focused on these aspects.

**Figure 1:**
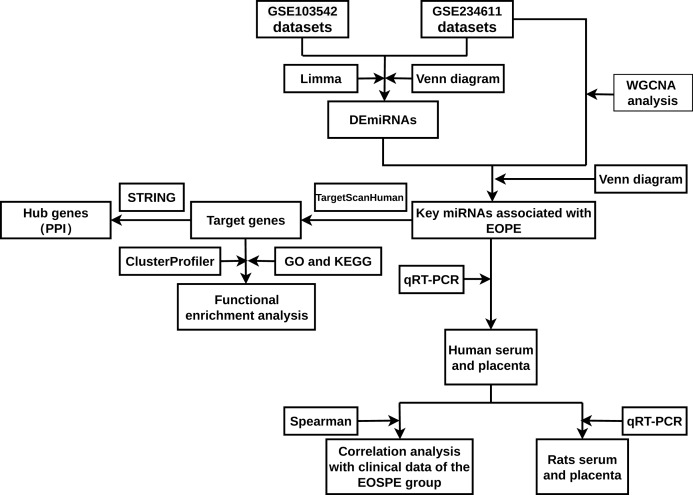
The flow diagram of the study.

With the ongoing advancement of genetic technologies, exploring key genes involved in the pathogenesis of PE from a genomic perspective has become an important direction for identifying new targets for PE treatment and prevention (Chen et al., 2025). This study aimed to screen for DEmiRNAs in PE patients based on the Expression Omnibus (GEO)database and comprehensively utilized various bioinformatics methods for their collation and analysis. The expression levels of the target miRNA were further validated in clinical specimens, aiming to provide a reference basis for the early diagnosis and prognostic assessment of EOSPE patients (Fig. 1).

## Materials and Methods

### Data collection

The miRNAs expression datasets from PE patients were searched and downloaded from the GEO database (https://www.ncbi.nlm.nih.gov/geo/) (Edgar, Domrachev & Lash, 2002). The GSE103542 dataset (Lykoudi et al., 2018) and the GSE234611 dataset (Cen et al., 2025), which are based on the GPL23980 and GPL24676 platforms, respectively, were ultimately selected. The GSE103542 dataset includes 16 PE placental tissue samples (11 from EOPE patients and five from late-onset preeclampsia (LOPE) patients) and eight normal placental tissue samples. The GSE234611 dataset includes 10 PE plasma samples (five from EOPE patients and five from LOPE patients) and seven normal plasma samples.

### Screening of differential miRNAs

In this study, R software (v4.2.1; R Core Team, 2024) was employed. The limma package (v3.52.2) was used to normalize and standardize the matrix data from PE patients. Differentially expressed miRNAs (DEmiRNAs) were identified using the threshold set at *P* < 0.05 and |Log2 fold change| ≥ 1. The molecules identified in the screen are merely candidate molecules and must be validated through subsequent experiments. The differential expression was visualized *via* a volcano plot, and a Venn diagram was utilized to identify the overlapping DEmiRNAs between the two datasets. The results were visualized with the R packages“ggplot2” (v3.4.4).

Furthermore, the Weighted Gene Co-expression Network Analysis (WGCNA) package (v1.72.5) was applied to the GSE234611 dataset to construct a miRNA co-expression network, thereby identifying biological modules associated with EOPE. The correlation coefficients between each module and different traits were calculated, and modules with a correlation coefficient ≥ 0.7 were selected. The miRNAs within these selected modules were then intersected with the known DEmiRNAs to screen for DEmiRNAs highly associated with EOSPE.

### Functional and pathway enrichment analysis of DEmiRNAs

For the DEmiRNAs screened above, target gene prediction was performed using the TargetScanHuman 8.0 online database (https://www.targetscan.org/vert_80/). Using R software (v4.2.1), the org.Hs.eg.db package (v4.2.1) was employed for ID conversion of the input molecule list. Subsequently, the clusterProfiler package (v4.4.4) was used to conduct Gene Ontology (GO) and Kyoto Encyclopedia of Genes and Genomes (KEGG) enrichment analyses on the target genes. The screening criterion was set at *P* < 0.05. The results were visualized with the R packages“ggplot2” (v3.4.4). These bioinformatics analyses were performed using Xiantao Academic (http://www.xiantao.love/).

### Construction of target gene protein-protein interaction network and screening of hub genes

The STRING database (https://string-db.org/) was used to construct a protein-protein interaction (PPI) network. The analysis species was set as “Homo sapiens”, with a confidence score threshold ≥ 0.7. Disconnected nodes in the network were hidden to construct and visualize the PPI network. CytoHubba, a Cytoscape plugin, was used to identify the top 10 hub genes within the PPI network.

### General clinical characteristics

This was a retrospective study. A total of 21 pregnant women with EOSPE who received prenatal examination and delivered at The Third Affiliated Hospital of Zhengzhou University from September 2023 to September 2024 were enrolled as the study group. During the same period, 16 normal pregnant women with full-term singleton pregnancies were selected as the control group (Cont group). All participants voluntarily took part in the study and provided written informed consent prior to enrollment. This study was approved by the Medical Ethics Committee of The Third Affiliated Hospital of Zhengzhou University (Approval No. 2023-092-02).

### Inclusion and exclusion criteria

#### Inclusion criteria

Cont group: Full-term delivery, healthy pregnant women without hypertensive disorders of pregnancy, diabetes, or other major diseases.

EOSPE group: The diagnostic criteria were based on the “Diagnosis and treatment of hypertension and pre-eclampsia in pregnancy: a clinical practice guideline in China (2020)” For pregnant women already diagnosed with PE, those with onset between 20 and 34 weeks of gestation presenting with acute onset and rapid progression were diagnosed with EOPE. Among PE patients, those meeting any of the following conditions—persistent hypertension, central nervous system symptoms, hepatic impairment, renal dysfunction, hematological abnormalities, or fetal compromise—were diagnosed with sPE.

#### Exclusion criteria

Pregnant women meeting any of the following criteria will be excluded from this study:

(1) Multiple pregnancy (number of fetuses ≥ 2);
(2) Pre-existing chronic conditions related to blood pressure, renal, thyroid function, or glucose metabolism abnormalities;
(3) Use of medications without medical approval during pregnancy, or a documented history of medication abuse;
(4) Conception achieved through assisted reproductive technology or ovulation induction;
(5) Previous diagnosis of autoimmune diseases, or presence of other diseases significantly affecting pregnancy outcomes;
(6) Inability to provide complete antenatal care records and pregnancy outcome data.

### Clinical data collection

The basic information collected during the study included: maternal age, gravidity and parity, gestational week at delivery, height, weight, blood pressure (systolic and diastolic), neonatal birth weight, umbilical artery blood flow S/D ratio, platelet count (PLT), alanine aminotransferase (ALT), aspartate aminotransferase (AST), creatinine (CREA), uric acid (UA), lactate dehydrogenase (LDH), prothrombin time (PT), thrombin time (TT), activated partial thromboplastin time (APTT), fibrinogen (FIB), D-dimer (DD), and other relevant data. Data with a missing rate exceeding 10% were excluded to ensure accuracy and completeness.

### Sample collection

Five milliliters of fasting venous blood were collected from pregnant women in the morning. The samples were left to stand at 4 °C for 30 min until natural coagulation occurred. Subsequently, the clotted blood was carefully transferred to a bench-top centrifuge and centrifuged at 1,500 × g and 4 °C for 10 min. The supernatant was collected, transferred to new sterile Eppendorf tubes, and stored at −80 °C for subsequent miRNA extraction.

After placental delivery during cesarean section, multiple tissue samples, each approximately 1 cm^3^ in volume, were promptly collected from the maternal side of the placenta and from areas near the umbilical cord on the maternal side. During sampling, care was taken to avoid areas with hemorrhage or calcification. After rinsing to remove residual blood, the samples were immediately frozen in liquid nitrogen and subsequently transferred to a −80 °C environment for storage. A total of 10 placental tissue samples were collected from both the Control and the EOSPE groups.

### Animal model

All animal experimental procedures were conducted in accordance with the ARRIVE guidelines and approved by the Animal Welfare and Research Ethics Committee of Zhengzhou University Laboratory Animal Center (Approval No. ZZU-LAC20240531[10]). Healthy female Sprague-Dawley (SD) rats (10–12 weeks old, weighing 200–250 g) were obtained from Henan Scebis Biotechnology Co., Ltd. (Henan, China) and housed under specific pathogen-free (SPF) conditions. The animals were housed under controlled conditions: temperature of 20–25 °C, humidity of 40–70%, and a 12-h light/dark cycle (lights on from 08:00 to 18:00). They were housed two per cage in a clean and quiet environment with free access to standard laboratory chow and autoclaved water.

Based on preliminary experimental data and predicted means and standard deviations for various designs and interventions, our analysis indicated that a sample size of 5–13 animals per group would yield approximately 0.75 statistical power. Twelve female SD rats were randomly and equally divided into two groups: the Cont group (*n* = 6) and the Lipopolysaccharide (LPS) group (*n* = 6). Female rats in each group were co-housed overnight with male SD rats at a 2:1 ratio. On the following morning, vaginal smears were taken from the female rats: the presence of a significant number of clear spermatozoa under microscopic examination confirmed the day as gestational day 0 (GD0). On gestational day 14 (GD14), rats in the LPS group received a slow tail vein injection of LPS solution (1 μg/kg, LPS dissolved in normal saline), while the control group received an equal volume of normal saline *via* tail vein injection. Both injections were completed within 10 min. Systolic blood pressure was measured and urine samples were collected from the pregnant rats on GD3, GD6, GD9, GD12, GD15, and GD18, respectively.

On gestational day 19 (GD19), pregnant rats were euthanized by intraperitoneal injection of 1% sodium pentobarbital (50 mg/kg). Peripheral blood was collected and centrifuged at 3500 × g for 10 min at 4 °C. Fetuses and placentas were then harvested immediately. Fetal body length and weight were recorded, and placental tissues were either fixed in 4% paraformaldehyde or snap-frozen and stored at −80 °C for subsequent analysis. At the end of the experiment, surviving male rats were euthanized with an overdose of sodium pentobarbital.

### Urine protein test

Urinary protein levels were measured on GD3, GD6, GD9, GD12, GD15, and GD18. Urine samples were collected over a 12-h period using metabolic cages. The urinary protein concentration for each group was determined using a BCA protein assay kit (Beyotime Biotechnology, Shanghai).

### Hematoxylin and eosin (HE) staining

Placental tissues, after fixation, paraffin embedding, and sectioning, were deparaffinized. Sequential staining with hematoxylin-eosin (HE) was performed, followed by dehydration through a gradient ethanol series, clearing with xylene, and mounting with neutral balsam. Histological changes were finally observed under an optical microscope.

### miRNAs extraction and reverse transcription

Total RNA, including miRNA, was extracted from human and rat serum and placental tissues using the miRcute miRNA Extraction and Separation Kit (DP501; Tiangen Biotech, Beijing, China). RNA purity and concentration were quantified using a Nanodrop spectrophotometer (Thermo Fisher Scientific, Waltham, MA, USA), with all samples exhibiting an A260/A280 ratio between 1.8 and 2.0. Reverse transcription and subsequent qRT-PCR were performed using the miDETECT A Track miRNA qRT-PCR Starter Kit (C10712-1; RiboBio, Guangzhou, China) according to the manufacturer’s protocol. The procedure consisted of three main steps: (1) poly (A) tailing, (2) reverse transcription, and (3) qPCR amplification. The qPCR reactions were initiated with a pre-denaturation step at 95 °C for 10 min, followed by 50 cycles of 95 °C for 5 s, 60 °C for 20 s, and 70 °C for 10 s. Each sample was run in technical triplicates in a 20 µL reaction volume. The specific primers for hsa-miR-431-5p (miRA1000554-1-100; RiboBio, Guangzhou, China), rno-miR-431-5p (miRACM001-12; RiboBio, Guangzhou, China), cel-miR-39-3p (miRA0000010-1-100; RiboBio, Guangzhou, China), and U6 (miRAN0002-1-100; RiboBio, Guangzhou, China), as well as the cel-miR-39-3p standard (miRB0000010-3-1; RiboBio, Guangzhou, China), were all obtained from RiboBio (Guangzhou, China). The 2−ΔΔCT method was used to calculate the relative expression of miR-431-5p, with cel-miR-39-3p serving as the internal reference for serum specimens and U6 small nuclear RNA (U6) as the internal reference for placental tissues. All PCR experiments and analyses adhered to the MIQE guidelines.

### Statistical analysis

Statistical analysis was performed using SPSS software (version 26.0). Normally distributed continuous data were expressed as mean ± standard deviation ( $\bar{\rm x}$ ± s), and comparisons between two groups were conducted using independent samples t-tests. Categorical data are presented as number (percentage) [*n* (%)], and intergroup comparisons were performed using the Chi-square test or Fisher’s exact test. Spearman correlation analysis was employed to assess the correlation between miR-431-5p expression levels and various clinical indicators. Multivariable logistic regression analysis, adjusting for maternal age and pre-pregnancy body mass index (BMI), was performed to evaluate the independent association between miR-431-5p and EOSPE. The diagnostic value of serum miR-431-5p for EOSPE was evaluated by plotting receiver operating characteristic (ROC) curves and calculating the area under the curve (AUC). The optimal cutoff value was determined by maximizing the Youden index. A *P* value < 0.05 was considered statistically significant.

## Results

### Acquisition of DEmiRNAs in preeclampsia

**Figure 2:**
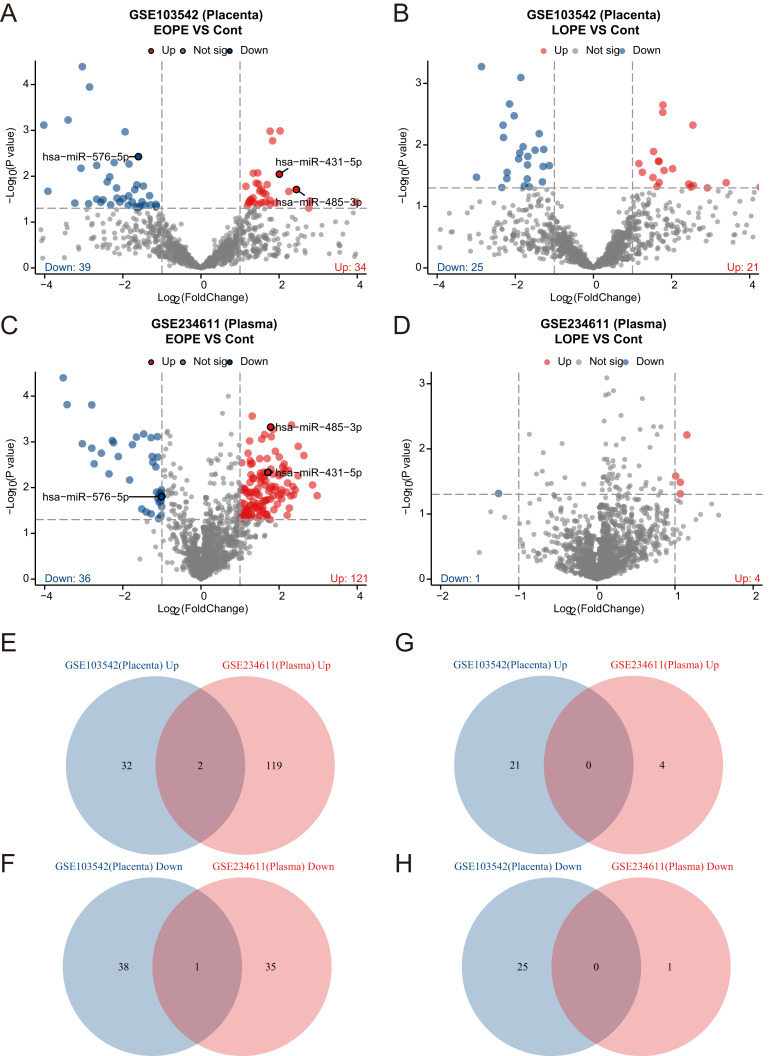
Identification of DEmiRNAs in PE based on the GEO database. (A–D) Volcano plots of DEmiRNAs from EOPE and LOPE comparisons in datasets GSE103542 (A, B) and GSE234611 (C, D). (E–H) Venn diagrams of overlapping up-regulated (E, G) and down-regulated (F, H) genes from EOPE (E, F) and LOPE (G, H) comparisons across both datasets. DEmiRNAs, differentially expressed miRNAs; PE, preeclampsia; GEO, Gene Expression Omnibus; EOPE, early-onset preeclampsia; LOPE, late-onset preeclampsia.

Analysis of the matrix data from GSE103542 and GSE234611 revealed that 73 DEmiRNAs were identified between the Cont group and the EOPE group in GSE103542, comprising 34 upregulated and 39 downregulated miRNAs (Fig. 2A). Similarly, 46 DEGs were found between the Cont group and the LOPE group in GSE103542, with 21 upregulated and 25 downregulated genes (Fig. 2B). In GSE234611, 157 DEGs were identified between the Cont group and the EOPE group, including 121 upregulated and 36 downregulated genes (Fig. 2C), whereas 5 DEGs were detected between the Cont group and the LOPE group, consisting of four upregulated and one downregulated gene (Fig. 2D). Venn diagram analysis of the intersecting DEGs from both datasets revealed three co-expressed genes: two were highly expressed (hsa-miR-431-5p and hsa-miR-485-3p) (Fig. 2E), and one was lowly expressed (hsa-miR-576-5p) (Fig. 2F). Notably, all three co-expressed genes originated from the DEGs between the Cont and EOPE groups, while no co-expressed genes were found between the Cont and LOPE groups across the two datasets (Figs. 2G, 2H). It should be noted that the differentially expressed molecules identified from the screening require further experimental validation.

### Identification of EOPE-associated miRNAs modules by WGCNA

**Figure 3:**
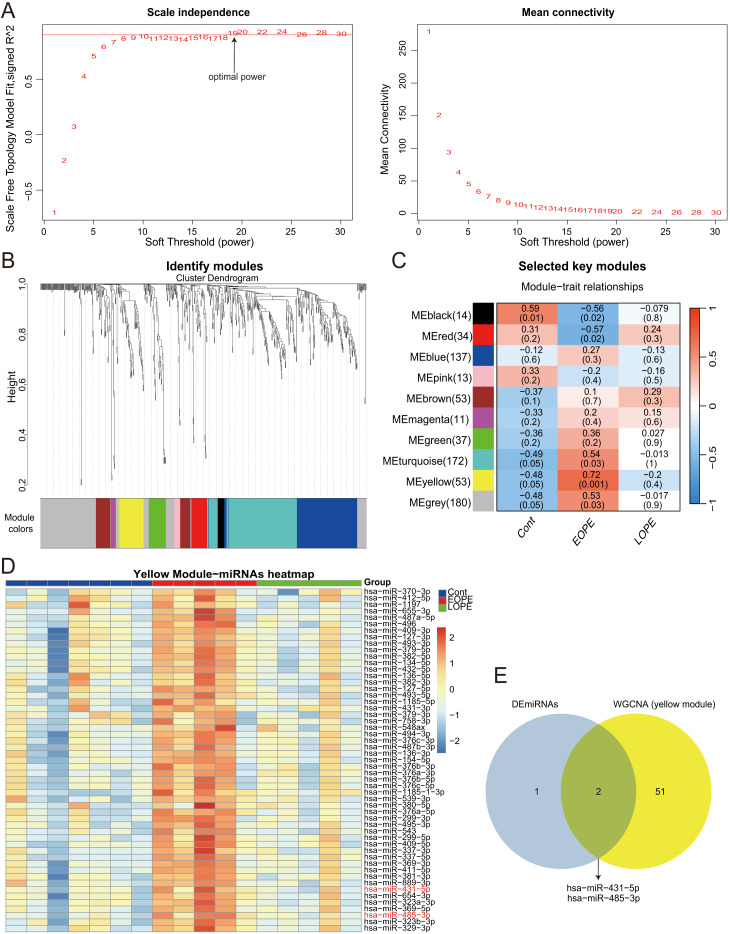
Identification of EOPE-related modules in WGCNA. (A) To determine the soft threshold (β) and assess the scale-free topology, gene-to-gene correlation calculations were conducted utilizing R^2^ values. The associated numbers indicate the selection of a suitable β. (B) The cluster dendrogram groups miRNAs based on their proximity, with miRNAs that are closer together clustered into distinct modules, each denoted by a unique color. Gray represents miRNAs that do not belong to any module. (C) The correlation heatmap illustrates the relationships between the identified modules and the experimental groups, with the yellow module highlighted as a key module due to its strong correlation (|r| > 0.70). (D) The heatmap presents the expression levels of miRNAs contained within the yellow module. (E) The Venn diagram visualizes the intersection of DEmiRNAs from the EOPE group and the miRNAs identified in the yellow module through WGCNA analysis, specifically hsa-miR-431-5p and hsa-miR-485-3p EOPE, early-onset preeclampsia; WGCNA, Weighted Gene Co-expression Network Analysis; DEmiRNAs, differentially expressed miRNAs.

We conducted a weighted gene co-expression network analysis (WGCNA) on the miRNA datasets to identify functionally significant modules associated with EOPE and LOPE. A scale-free co-expression network was successfully constructed, revealing multiple distinct modules through hierarchical clustering (Figs. 3A, 3B). Among these, the yellow module (Fig. 3C), which consisted of 53 miRNAs (as shown in the heatmap, Fig. 3D), exhibited a strong correlation with EOPE (r = 0.72, *P* = 0.001). Notably, both hsa-miR-431-5p and hsa-miR-485-3p were identified as DEmiRNAs in EOPE compared with the control group, and they were also closely associated with EOPE (Fig. 3E). This suggests that both miRNAs may play significant roles in the occurrence and progression of EOPE.

### GO and KEGG pathway enrichment analysis of DEmiRNAs

**Figure 4:**
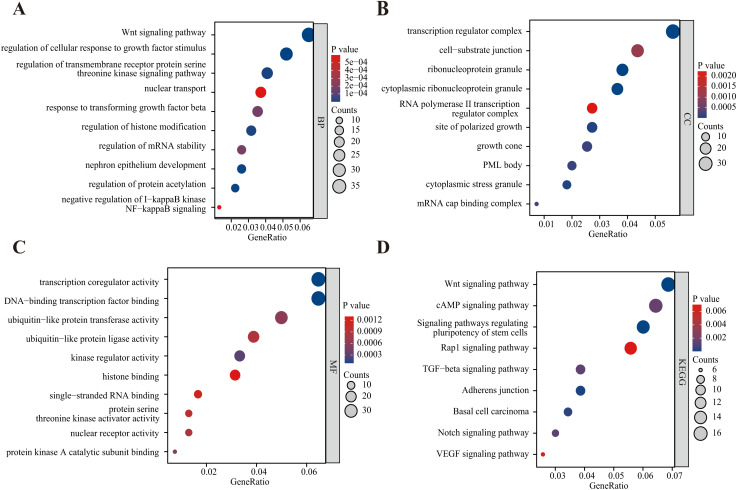
GO and KEGG enrichment analysis of differentially expressed miRNAs. (A–C) GO functional annotation of differentially expressed miRNAs. (D) KEGG pathway enrichment analysis. DEmiRNAs, differentially expressed miRNAs; GO, Gene Ontology; KEGG, Kyoto Encyclopedia of Genes and Genomes.

In-depth GO and KEGG pathway enrichment analyses were performed on the target genes predicted by the TargetScanHuman 8.0 online database. GO enrichment analysis revealed that the biological processes (BP) of the differentially expressed genes were enriched in processes such as the Wnt signaling pathway, cellular response to growth factor stimulus, nuclear transport, regulation of histone modification, regulation of mRNA stability, and negative regulation of the I-κB/NF-κB signaling pathway (Fig. 4A). The cellular components (CC) were mainly enriched in transcriptional regulatory complexes (RNA polymerase II transcription regulator complex), cell-substrate junctions, ribonucleoprotein granules (including cytoplasmic stress granules), and subcellular structures such as PML bodies (Fig. 4B). The molecular functions (MF) were predominantly associated with transcription co-regulator activity, DNA-binding transcription factor binding, ubiquitin-like protein transferase activity and ligase activity, kinase regulator activity, histone binding, single-stranded RNA binding, and protein kinase A catalytic subunit binding (Fig. 4C). KEGG pathway analysis indicated that the differentially expressed genes were mainly involved in signaling pathways such as Wnt, cAMP, TGF-β, Rap1, and the Regulation of pluripotency of stem cells (Fig. 4D).

### Construction of the PPI network of target genes and screening of hub genes

**Figure 5:**
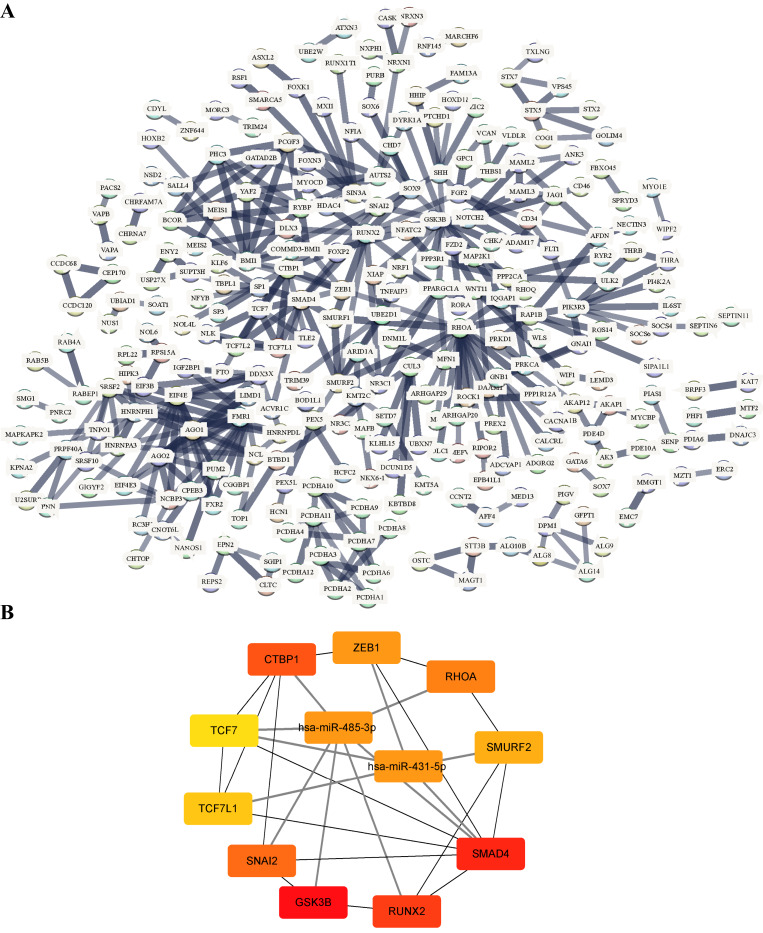
Construction of the target gene PPI network and identification of hub genes. (A) PPI network of target genes constructed using the STRING online database. (B) PPI network of Hub genes constructed using Cytoscape software (version 3.10.3). PPI, protein-protein interaction.

The interaction data for target genes of differentially expressed miRNAs were visualized using the STRING online database, and a PPI network was constructed (Fig. 5A). The cytoHubba plugin in Cytoscape software (version 3.10.3) was used to identify the top 10 hub genes based on protein interaction network ranking, namely GSK3B, RUNX2, SMAD4, SMURF2, RHOA, ZEB1, CTBP1, SNAI2, TCF7, and TCF7L1 (Fig. 5B).

### Expression of miR-431-5p in serum and placenta of EOSPE and control groups and its diagnostic value for EOSPE

**Figure 6:**
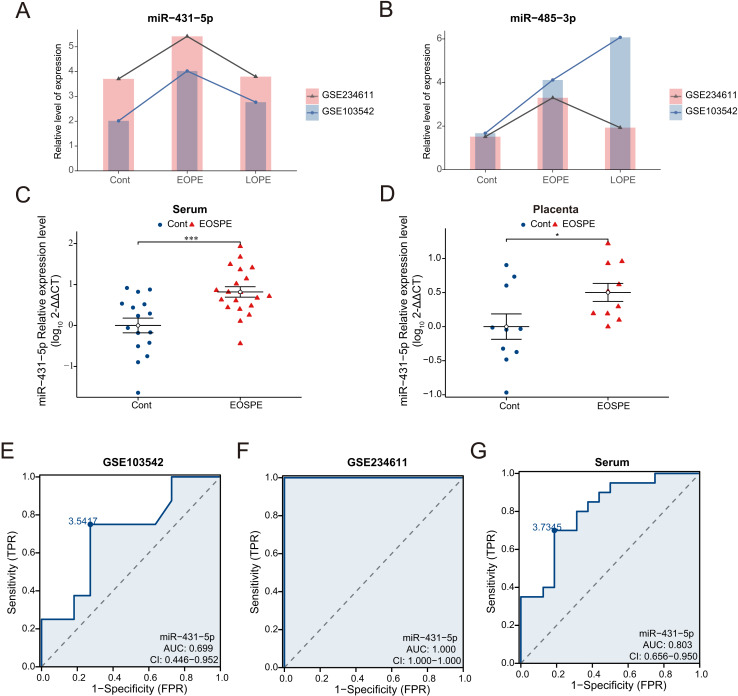
Expression of miR-431-5p in serum and placental tissues of EOSPE patients and ROC curves. (A, B) Screening of candidate miRNAs (miR-431-5p and miR-485-3p) in GEO datasets (GSE103542, GSE234611). (C, D) miR-431-5p expression in clinical EOSPE samples: (C) serum and (D) placental tissue. (E–G) Diagnostic performance (ROC curves) of miR-431-5p in (E) GSE103542, (F) GSE234611, and (G) clinical serum. *P < 0.05; ***P < 0.001. EOSPE, Early-onset severe preeclampsia; ROC, Receiver Operating Characteristic; GEO, Gene Expression Omnibus.

We analyzed the raw expression levels of miR-431-5p and miR-485-3p in both the GSE103542 and GSE234611 datasets. Our analysis revealed that miR-431-5p exhibited a consistent expression pattern across both datasets: compared with the Cont group, its expression was elevated in both the EOPE and LOPE groups, with significantly higher levels in the EOPE group than in the LOPE group (Fig. 6A). In contrast, the expression trend of miR-485-3p was inconsistent across the two datasets (Fig. 6B). Given that this study aimed to identify potential biomarkers for EOSPE, we subsequently focused our investigation on miR-431-5p.

We used reverse transcription-quantitative polymerase chain reaction (RT-qPCR) to detect miR-431-5p expression levels in the serum of pregnant women from the EOSPE and Cont groups. We found that miR-431-5p expression was significantly higher in the EOSPE group than in the Cont group (*P* < 0.05), which was consistent with our aforementioned bioinformatics results (Fig. 6C). We further validated miR-431-5p expression in the placental tissues of patients from the EOSPE and Cont groups. Similarly, the expression of miR-431-5p was significantly higher in the EOSPE group compared with the Cont group (Fig. 6D).

To further evaluate the predictive value of miR-431-5p for patients with EOSPE, we assessed its diagnostic performance in distinguishing the EOPE group from the Cont group using the GSE103542, GSE234611, and our human serum dataset. The optimal cut-off value for the ROC curve was determined by the Youden index. In the GSE103542 dataset, the area under the curve (AUC) of miR-431-5p was 0.699 (95% CI [0.446–0.952]), with a sensitivity of 75% and a specificity of 72.2%. The relatively wide confidence interval (width: 0.506) might be attributed to the small sample size of this dataset. In the GSE234611 dataset, the AUC was 1.000 (95% CI [1.000–1.000]), with both sensitivity and specificity of 100% (Fig. 6F). Notably, the small sample size and complete separation of expression values between the two groups in this dataset precluded a reliable estimation of the confidence interval; therefore, this result should be considered exploratory and at risk of overfitting. In our human serum samples, the AUC was 0.803 (95% CI [0.656–0.950]), with a sensitivity of 70% and a specificity of 81.3% (Fig. 6G). The confidence interval was also relatively wide (width: 0.294), but its lower bound exceeded 0.65, suggesting that miR-431-5p has promising diagnostic potential for EOSPE and may serve as a candidate biomarker.

### Analysis of clinical characteristics of pregnant women in the cont group and EOSPE group

Significant differences were observed between the Cont group and the EOSPE group in the following clinical parameters: gestational age at delivery, BMI, systolic blood pressure (SBP), diastolic blood pressure (DBP) neonatal birth weight, umbilical artery blood flow systolic/diastolic (S/D) ratio, incidence of fetal growth restriction, as well as levels of alanine transaminase (ALT), aspartate transaminase (AST), creatinine (CREA), uric acid (UA), lactate dehydrogenase (LDH), and Prothrombin Time (PT) (all *P* < 0.05; Table 1).

**Table 1:** Comparison of clinical characteristics between the two groups.

Variable	Control group (*n* = 16)	EOSPE group (*n* = 20)	*P*-value
Age (years)	33.00 ± 3.98	33.76 ± 4.02	0.570
Pregnancy number (times)	2.31 ± 1.92	2.67 ± 1.43	0.229
Number of births (times)	0.69 ± 0.87	0.95 ± 0.92	0.349
Gestational age (weeks)	39.51 ± 0.97	30.86 ± 2.18	<0.001
BMI (kg/m^2^)	27.02 ± 3.58	30.23 ± 5.26	0.018
SBP (mmHg)	114.81 ± 8.37	156.48 ± 12.04	<0.001
DBP (mmHg)	67.44 ± 8.12	101.24 ± 8.16	<0.001
Neonatal birth weight (g)	3,340.00 ± 457.55	1,213.30 ± 396.85	<0.001
S/D ratio	2.37 ± 0.47	3.55 ± 0.80	<0.001
Fetal growth restriction	No 16 (43.2%)	No 12 (32.4%)	0.003
	Yes 0 (0%)	Yes 9 (24.3%)	
PLT (10^9^/L)	209.94 ± 45.58	176.05 ± 65.06	0.085
ALT (U/L)	14.88 ± 2.39	31.58 ± 19.66	0.005
AST (U/L)	20.88 ± 2.68	30.12 ± 12.36	0.018
CREA (μmol/L)	42.87 ± 7.10	60.79 ± 12.71	<0.001
UA (μmol/L)	281.43 ± 102.50	448.90 ± 91.00	<0.001
LDH (U/L)	166.81 ± 16.69	274.99 ± 102.08	<0.001
PT	10.07 ± 2.28	9.64 ± 0.72	<0.001
TT	14.84 ± 0.91	15.48 ± 1.73	0.158
APTT	27.13 ± 2.26	29.08 ± 4.06	0.133
FIB (g/L)	4.03 ± 0.43	3.90 ± 0.69	0.510
DDI (mg/L)	0.73 ± 0.53	1.07 ± 1.35	0.890

**Note:**

BMI, Body Mass Index; SBP, Systolic Blood Pressure; DBP, Diastolic Blood Pressure; S/D ratio, Umbilical Artery Blood Flow S/D Ratio; PLT, Platelet Count; ALT, Alanine Aminotransferase; AST, Aspartate Aminotransferase; CREA, Creatinine; UA, Uric Acid; LDH, Lactate Dehydrogenase; PT, Prothrombin Time; TT, Thrombin Time; APTT, Activated Partial Thromboplastin Time; FIB, Fibrinogen; DD, D-dimer.

### Correlation analysis of miR-431-5p with clinical data

We further analyzed the correlation between miR-431-5p expression levels and specific clinical indicators. The results showed that miR-431-5p expression was significantly positively correlated with umbilical artery blood flow S/D ratio, SBP, DBP, ALT, CREA, UA, and LDH, and was statistically significantly negatively correlated with both gestational age at delivery and neonatal birth weight (all *P* < 0.05; Table 2).

**Table 2:** Correlation analysis between miR-431-5p and clinical parameters in the EOSPE group.

miR-431-5p	r (Correlation coefficient)	*P*-value
Age	0.008	0.965
Pregnancy number	−0.311	0.065
Number of births	0.269	0.113
S/D ratio	0.457	0.01
Gestational age	−0.562	<0.001
Neonatal birth weight	−0.503	0.002
SBP	0.395	0.017
DBP	0.41	0.013
PLT	0.035	0.841
ALT	0.36	0.031
AST	0.191	0.263
CREA	0.371	0.027
UA	0.389	0.02
LDH	0.399	0.018
PT	−0.298	0.077
TT	−0.066	0.701
APTT	−0.092	0.595
FIB	−0.03	0.862
DDI	−0.011	0.948
BMI	0.277	0.102

**Note:**

EOSPE, Early-onset severe preeclampsia; SBP, Systolic Blood Pressure; DBP, Diastolic Blood Pressure; S/D ratio, Umbilical Artery Blood Flow S/D Ratio; PLT, Platelet Count; ALT, Alanine Aminotransferase; AST, Aspartate Aminotransferase; CREA, Creatinine, UA, Uric Acid; LDH, Lactate Dehydrogenase; PT, Prothrombin Time; TT, Thrombin Time; APTT, Activated Partial Thromboplastin Time; FIB, Fibrinogen; DD, D-dimer; BMI, Body Mass Index.

### Multivariable logistic regression analysis

To evaluate the independent association between miR-431-5p expression and the risk of EOSPE, we performed multivariable logistic regression analysis. Collinearity diagnostics indicated no significant multicollinearity among the covariates (variance inflation factor < 5 for all variables). After stepwise adjustment for age and BMI, miR-431-5p remained significantly and positively associated with the occurrence of EOSPE, suggesting that it may be an independent factor for EOSPE (OR = 1.31, 95% CI [1.01–1.71], *P* = 0.047; Table 3).

**Table 3:** Multivariable logistic regression analysis of the association between miR-431-5p expression and EOSPE risk.

Variables	Model 1		Model 2		Model 3	
	OR (95% CI)	*P*	OR (95% CI)	*P*	OR (95% CI)	*P*
**miR-431-5p**	**1.32 [1.01–1.74]**	**0.043**	**1.33 [1.01–1.75]**	**0.041**	**1.31 [1.01–1.71]**	**0.047**

**Note:**

OR, Odds Ratio; CI, Confidence Interval. Model1: Crude Model2: Adjust: Age Model3: Adjust: Age, BMI

### Evaluation of the PE rat model and the expression of miR-431-5p in serum and placenta of preeclamptic rats

**Figure 7:**
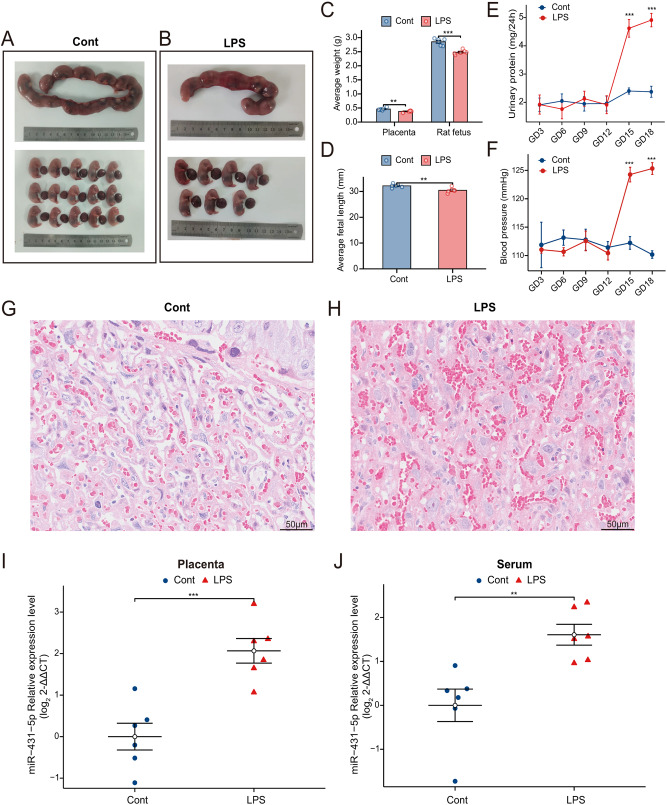
Evaluation of the PE rat model and expression of miR-431-5p in serum and placental tissues of PE rats. (A, B) Gross morphological features of uteri, fetuses, and placentas. (C, D) Biometric measurements of (C) weights and (D) fetal length. (E, F) Disease progression markers: (E) proteinuria and (F) hypertension. (G, H) Placental histology (HE staining). (I, J) miR-431-5p expression in (I) placenta and (J) serum. **P < 0.01; ***P < 0.001. PE, Preeclampsia; HE, Hematoxylin and Eosin.

To exclude confounding variables such as ethnicity and environment, and to substantiate the universal and critical role of miR-431-5p in the pathological process of PE, we validated its expression in an LPS-induced rat model of PE. Compared with the Cont group, the LPS group showed significant reductions in placental weight, fetal weight, and fetal body length (Figs. 7C, 7D). Blood pressure and urinary protein levels were significantly elevated in the LPS group (Figs. 7E, 7F). HE staining revealed obvious inflammatory cell infiltration in the placental tissues of the LPS group compared with the Cont group (Figs. 7G, 7H). These results confirmed the successful establishment of the PE model. RT-qPCR analysis demonstrated that the expression level of miR-431-5p in both serum and placental tissues of the LPS group was significantly higher than that in the Cont group (Figs. 7I, 7J).

## Discussion

Currently, the most widely used clinical biomarker for PE is the sFlt-1/PlGF ratio, which has good predictive value (Kutllovci Hasani, Ajeti & Goswami, 2025). However, it has certain limitations, including limited specificity for EOSPE, an inability to distinguish between PE subtypes, and relatively high detection costs. Other biomarkers, such as PAPP-A and PP13, are influenced by gestational age and maternal BMI (Lakiang et al., 2021). Therefore, further exploration of biomarkers specifically tailored to early-onset preeclampsia is warranted.

In this study, by integrating bioinformatics and clinical validation approaches, we found that miR-431-5p is persistently upregulated in EOSPE. Our research revealed that, compared to the Cont group, miR-431-5p was significantly elevated in both the serum and placenta of EOSPE patients, and serum miR-431-5p exhibited significant diagnostic potential for EOSPE. Furthermore, the expression level of miR-431-5p was correlated with key clinical parameters of disease severity. The observed upregulation of miR-431-5p in the LPS-induced PE rat model further supports its association with the disease.

EOSPE is a particularly critical subtype of PE (Peguero et al., 2023). Current research primarily focuses on EOPE as a whole, whereas investigations into the specific miRNA expression profiles, diagnostic value, and regulatory mechanisms of the EOSPE subtype remain relatively scarce. Our research strategy aims to overcome the limitations of previous studies and to identify reliable and effective early biomarkers for EOSPE. miRNAs are key regulators of cellular biological processes, and some miRNAs play crucial roles in modulating trophoblast function and inflammatory responses in PE (Asadi-Tarani et al., 2020; Chiang, Seow & Chen, 2024; Wang, Li & Zhao, 2022; Zhou et al., 2023). Existing studies have reported elevated expression of miR-146a-5p in the placentas of PE patients. miR-146a-5p mediates trophoblast proliferation and invasion by regulating Wnt2 expression (Peng et al., 2021). miR-138 participates in the pathogenesis of PE by targeting RELA, thereby influencing NF-κB signaling, and inducing inflammatory responses and oxidative stress in trophoblasts (Yin et al., 2021). In this study, we performed differential analysis of miRNA expression profiles in both placental and serum specimens and combined with WGCNA to further screen modules strongly associated with EOPE, ultimately identifying DEmiRNAs. This multi-dimensional screening strategy further ensures the reliability of the candidate molecules.

We performed GO and KEGG analyses on the screened DEmiRNAs. Our analysis revealed that these genes were significantly enriched primarily in signaling pathways such as Wnt, cAMP, TGF-β, and I-κB/NF-κB. Previous studies have found that abnormal activation of the Wnt pathway can inhibit trophoblast cell migration, leading to inadequate remodeling of the uterine spiral arteries, which further contributes to the onset of PE (Fu et al., 2023; Liu et al., 2024; Zhang et al., 2018; Zhu & Chen, 2023). Our study further revealed that the Wnt pathway might be regulated by miR-431-5p to promote the pathogenesis and injury of EOSPE. The TGF-β signaling pathway is involved in various cellular activities such as cell growth, differentiation, and apoptosis, and can induce cell cycle arrest in early cancer cells. It has been reported that the TGF-β signaling pathway can regulate trophoblast cell differentiation and invasion, thereby promoting the pathogenesis of PE (Haider et al., 2022; Li et al., 2021). The cAMP pathway is associated with vascular smooth muscle relaxation, and its dysfunction may exacerbate elevated blood pressure (Duan et al., 2021; Li et al., 2022). Furthermore, we identified the top 10 hub genes from the PPI network, such as GSK3B, RUNX2, SMAD4, RHOA, ZEB1, and TCF7. Overactivation of GSK3B inhibits the Wnt pathway, leading to increased β-catenin degradation, which subsequently reduces the invasive capacity of trophoblasts, causing placental ischemia and hypoxia. Concurrently, GSK3B promotes the expression of pro-inflammatory factors such as IL-6, TNF-α, and IL-1β, exacerbating systemic inflammatory responses and further triggering PE (Feng et al., 2025; Zhang et al., 2024). SMAD4 is a core factor in the TGF-β signaling pathway. Its expression is significantly downregulated in the placental tissue of PE patients and shows a negative correlation with miR-34a-5p, which is highly expressed in PE (Xue et al., 2019). RHOA is a key effector molecule downstream of the S1P-S1PR2 signaling axis. It regulates YAP subcellular localization and transcriptional activity by activating ROCK-mediated actin polymerization, thereby maintaining the invasion and migration capabilities of trophoblasts. Reduced RHOA activity leads to impaired trophoblast function and placental developmental defects, ultimately contributing to the occurrence and development of PE (Liao et al., 2022). Most of these genes are associated with the Wnt pathway, inflammatory responses, and trophoblast function, providing a reference for our subsequent research.

We further integrated clinical samples to systematically investigate the expression characteristics and clinical significance of miR-431-5p in the serum and placental tissues of EOSPE patients. Our preliminary analysis identified miR-431-5p and miR-485-3p as highly associated with EOPE. However, we found an inconsistent expression trend for miR-485-3p between the two datasets: its upregulation in the LOPE group was significantly higher than that in the EOPE group in the GSE103542 dataset. In contrast, miR-431-5p was stably and highly expressed in the EOSPE samples across both datasets. Consequently, we ultimately selected miR-431-5p as the core focus for subsequent research. Our results demonstrated that miR-431-5p is significantly highly expressed in both the serum and placental tissues of EOSPE patients. This finding is not only highly consistent with our bioinformatics predictions but also aligns with discoveries from researchers such as Lykoudi et al. (2018) and Yang & Meng (2019), thereby repeatedly validating the association between miR-431-5p and PE across different populations and studies. However, their work primarily focused on the expression of miR-431-5p in human placental tissue. Our study systematically conducted a cross-sample integrated analysis and confirmed the consistent upregulation of miR-431-5p in both placenta and serum from EOSPE patients. This finding suggests that the serum level of miR-431-5p may reflect placental pathological conditions and indicates its potential as a non-invasive candidate biomarker. Furthermore, ROC curve analysis showed that miR-431-5p has potential diagnostic value for EOSPE, suggesting that it might be useful for the early identification of EOSPE in future studies.

To further explore whether this marker might reflect the severity of EOSPE and perinatal outcomes, we first investigated its association with key clinical parameters using Spearman correlation analysis across all study participants. The expression level of miR-431-5p showed correlations with both disease severity and perinatal outcomes. To further assess its independent diagnostic value, we subsequently performed multivariable logistic regression analysis. After stepwise adjustment for potential confounders including age and BMI, miR-431-5p remained positively associated with the occurrence of EOSPE, suggesting that it may be an independent factor for EOSPE.

In terms of maternal outcomes, our analysis indicated that miR-431-5p was positively correlated with SBP, DBP, UA, and LDH. Hypertension is a core clinical feature of EOSPE (Lan et al., 2025; Wang et al., 2021; Zhao et al., 2017), raising the possibility that miR-431-5p may be involved in the regulation of vascular contractile function. Elevated UA is associated with renal impairment (Goldberg et al., 2021), and LDH is a marker of tissue hypoxia and injury (Zhou, Xiao & Yang, 2023). These findings suggest that miR-431-5p expression may be associated with maternal organ involvement. Regarding fetal outcomes, we observed that miR-431-5p was negatively correlated with gestational age at delivery and newborn birth weight, and positively correlated with the umbilical artery S/D ratio. An elevated umbilical artery S/D ratio is considered an indicator of inadequate placental perfusion (Lin et al., 2025). Shortened gestational age and low birth weight are major adverse fetal outcomes associated with EOSPE. These results suggest that miR-431-5p expression levels may be associated with fetal and placental function. Similarly, other researchers have reported that increased expression of miR-200a-3p in patients with severe preeclampsia is associated with adverse pregnancy outcomes (Lin et al., 2025), which shares similarities with our results. Our findings indicate that miR-431-5p expression levels may, to some extent, reflect disease severity and the risk of adverse prognosis in EOSPE. As a potential independent factor for EOSPE, it may offer additional information beyond traditional biomarkers and could serve as a reference for disease stratification and timing of intervention, although further validation is needed.

Based on the preceding enrichment analysis results and to further substantiate whether the critical role of miR-431-5p in the pathogenesis of PE possesses cross-species universality, we ultimately employed LPS to establish a PE rat model. We then validated the expression level of miR-431-5p in the serum and placental tissues of these model rats. We found that the expression of miR-431-5p in both the serum and placental tissues of the model rats was consistent with that in human samples. This finding is consistent with the clinical discovery and helps exclude confounding factors such as ethnicity and environment, providing additional support for a potential role of miR-431-5p in the pathological process of PE.

On this basis, miR-431-5p, as a circulating microRNA, has the potential to serve as a useful complement to existing biomarkers. Based on the preliminary data from this study and its molecular characteristics, its potential advantages are mainly reflected in the following aspects.

(1) Subtype specificity: Most existing biomarkers target preeclampsia as a whole, whereas this study focused on EOSPE, suggesting that miR-431-5p may fill the gap for this severe subtype. (2) Pathological relevance and stratification value: sFlt-1/PlGF mainly reflects anti-angiogenic imbalance, whereas miR-431-5p is predicted to regulate Wnt, TGF-β, and NF-κB pathways and key genes (*e.g*., GSK3B, SMAD4, RHOA), linking it to trophoblast invasion, inflammation, and vascular function. Its expression correlates with maternal indicators (blood pressure, uric acid, LDH) and perinatal outcomes (gestational age, birth weight, umbilical artery S/D ratio), suggesting potential for diagnosis, severity assessment, and risk stratification—addressing the lack of dynamic stratification in current markers. (3) Detection convenience: miR-431-5p can be measured in serum obtained through a minimally invasive blood draw. This approach uses qPCR, a mature, simple, and low-cost technique, which theoretically facilitates large-scale screening and use in primary care.

In summary, miR-431-5p, as a serum miRNA candidate biomarker associated with disease severity, has the potential to serve as a valuable complement to the existing PE biomarker panel and may be particularly useful for the early identification and disease assessment of the high-risk EOSPE subtype.

Although this study preliminarily suggests the diagnostic value of miR-431-5p in EOSPE, several limitations should be acknowledged. First, this was a single-center study with a limited sample size, which may affect the generalizability and stability of the conclusions. Due to the small sample size, the confidence intervals in the ROC analysis were relatively wide. Furthermore, formal false discovery rate (FDR) correction was not applied for multiple comparisons because of the limited sample size, and the possibility of false positive findings cannot be excluded. Therefore, the reported diagnostic performance estimates should be considered exploratory and require validation in larger, independent cohorts. Future studies should adopt multi-center, prospective cohort designs with larger sample sizes to validate the diagnostic accuracy reported here and to obtain more precise confidence intervals, and to perform FDR correction to enhance the reliability of the findings. Second, the current exploration of the potential functions of miR-431-5p relies primarily on bioinformatic predictions and correlation analyses. Direct functional experimental evidence—such as trophoblast migration/invasion assays following miR-431-5p overexpression or knockdown, luciferase reporter assays, and *in vivo* intervention studies—is lacking to elucidate its specific molecular mechanisms. In future studies, we plan to perform these *in vitro* and *in vivo* experiments to establish whether miR-431-5p plays a causal role in EOSPE pathogenesis. Third, the LPS-induced rat model used in this study mainly mimics inflammatory pathways rather than placental ischemia, which limits its ability to fully replicate human EOSPE. In future studies, we will establish additional animal models (*e.g*., the RUPP model) to further investigate the functional impact of miR-431-5p and to better understand its role in the pathogenesis of EOSPE. Fourth, we only evaluated the diagnostic performance of miR-431-5p alone and did not perform combined analyses with other clinical or biochemical markers. Future studies should integrate miR-431-5p with conventional biomarkers to determine whether it can improve the diagnostic rate of EOSPE. Fifth, this study did not include a spontaneous preterm birth control group or longitudinal sample collection. Since EOSPE is inherently associated with preterm delivery, the observed molecular alterations may be partly confounded by gestational age or preterm birth itself. Consequently, we cannot yet fully disentangle the molecular features specific to EOSPE from the effects of gestational age. Future studies should incorporate a spontaneous preterm birth control group and longitudinal sampling to verify the specificity of miR-431-5p for EOSPE.

## Conclusions

In this exploratory study, we observed that miR-431-5p was upregulated in both serum and placental tissues of patients with EOSPE. Its expression level is associated with disease severity and adverse perinatal outcomes, and it shows potential diagnostic value. Furthermore, bioinformatic analysis suggested that miR-431-5p may be involved in the pathological process of EOSPE, possibly through regulating the Wnt pathway and hub genes such as GSK3B and SMAD4. Taken together, miR-431-5p shows promise as a candidate biomarker for EOSPE, warranting further investigation.

## Supplemental Information

10.7717/peerj.21591/supp-1 Supplemental Information 1 Relative expression of miR-431-5p in human serum and placental tissue.

10.7717/peerj.21591/supp-2 Supplemental Information 2 RT-qPCR raw data. Relative expression levels of miR-431-5p in rat serum and placental tissue

10.7717/peerj.21591/supp-3 Supplemental Information 3 GSE234611 dataset.

10.7717/peerj.21591/supp-4 Supplemental Information 4 GSE103542 dataset.

10.7717/peerj.21591/supp-5 Supplemental Information 5 Clinical data.

10.7717/peerj.21591/supp-6 Supplemental Information 6 Systolic blood pressure data.

10.7717/peerj.21591/supp-7 Supplemental Information 7 Fetal growth data.

10.7717/peerj.21591/supp-8 Supplemental Information 8 Rat placenta fetal weight data.

10.7717/peerj.21591/supp-9 Supplemental Information 9 Urine protein data.

10.7717/peerj.21591/supp-10 Supplemental Information 10 R code. WGCNA code.

10.7717/peerj.21591/supp-11 Supplemental Information 11 ARRIVE checklist.

10.7717/peerj.21591/supp-12 Supplemental Information 12 MIQE checklist.

10.7717/peerj.21591/supp-13 Supplemental Information 13 Xiantao Academic Methodology. The core bioinformatics analyses in this study, including “differential analysis” and “functional and pathway enrichment analyses of differentially expressed miRNAs,” were performed using an online bioinformatics analysis platform named “Xiantao Academic” https://www.xiantao.love/. As a commercialized web-based tool, it operates through parameter selection and menu clicks, and its analysis process does not generate or provide downloadable R or Python script code. We have clearly described the use of this tool in Lines 139–140 of the manuscript. To compensate for the inability to provide traditional code and ensure the methodological transparency and reproducibility of the study, we have uploaded the methodological section of the platform (as applied in our analysis). Screenshots are included to demonstrate the platform’s interface together with their corresponding English translations.

## Additional Information and Declarations

### Data Availability

The following information was supplied regarding data availability:

The code and raw data are available in the Supplemental Files.
