# Supplementary material for: Identifying miR-431-5p as a promising candidate biomarker for early-onset severe preeclampsia through integrated bioinformatic and clinical analyses: a retrospective study
Source: PeerJ. 2026 Aug 11;14:e21591. doi: 10.7717/peerj.21591 (PMC13475390; doi:10.7717/peerj.21591)
Supplement: Supplementary file 2 [file peerj-14-21591-s013.pdf]

Screening of Differential miRNAs

差异分析-limma

差异分析-limma: 基于limma包标准差异分析流程, 对数据进行两组差异分析

Differential Analysis – limma

Differential Analysis - limma: Based on the standard differential analysis workflow of the limma package, perform two-group differential analysis on the data.

方法学

软件: R (4.2.1)版本

R包: limma[3.52.2]

处理过程:
· 使用limma包, 根据样本分组对数据进行标准组间差异分析

Methodology

Software: R version 4.2.1

R package: limma [3.52.2]

Processing steps:

Standard differential expression analysis between groups was performed using the limma package according to the sample grouping.

方法学

软件: R (4.2.1)版本

R包: ggplot2[3.4.4]

处理过程:
· 利用ggplot2包对差异分析结果进行可视化

补充说明:
· (阈值) logFC: 1
· (阈值) p: 0.05

Methodology

Software: R version 4.2.1

R package: ggplot2 [3.4.4]

Processing steps:

Visualization of differential expression analysis results was performed using the ggplot2 package.

Supplementary notes (thresholds):

logFC: 1

p-value: 0.05

Functional and Pathway Enrichment Analysis of DEmiRNAs

GOKEGG富集分析

GOKEGG: 拿一堆有功能注释的分子与所有有功能注释的分子去比较（超几何分布检验），确定那一堆中都涉及了哪些功能作用或者通路。
过程: 输入的分子列表 →(内部)→ 转成Entrez ID →(内部)→ 在GOKEGG的库进行超几何分析 → 获得分析结果

GO/KEGG Enrichment Analysis

GO/KEGG: Compares a set of molecules with functional annotations to all molecules with functional annotations using a hypergeometric distribution test, to identify which functional terms or pathways are overrepresented in the input set.

Procedure: Input molecule list → (internally) convert to Entrez IDs → (internally) perform hypergeometric analysis against GO/KEGG databases → obtain enrichment results.

方法学

软件: R (4.2.1)版本

R包: clusterProfiler[4.4.4]

处理过程:
· 对输入的分子列表进行ID转换后，用clusterProfiler包进行富集分析

补充说明:
· 物种: 人源(Homo sapiens)
· ID转换库(包): org.Hs.eg.db

Methodology

Software: R version 4.2.1

R package: clusterProfiler [4.4.4]

Processing steps:

After ID conversion of the input molecule list, enrichment analysis was performed using the clusterProfiler package.

Supplementary notes:

Species: Homo sapiens

ID conversion database/package: org.Hs.eg.db

方法学

软件: R (4.2.1)版本

R包: ggplot2[3.4.4]

处理过程:
· 使用ggplot2包对富集分析结果进行可视化

Methodology

Software: R version 4.2.1

R package: ggplot2 [3.4.4]

Processing steps:

Visualization of enrichment analysis results was performed using the ggplot2 package.
